# Hajdu-Cheney Syndrome with Fatal Progressive Basilar Invagination: Illustrative Case

**DOI:** 10.3390/children12091173

**Published:** 2025-09-02

**Authors:** Alim Emre Basaran, Matthias Krause, Johannes Wach, Erdem Güresir, Ulf Nestler, Christoph-Eckhard Heyde, Nicolas Heinz von der Höh

**Affiliations:** 1 Department of Neurosurgery, University Hospital Leipzig, 04103 Leipzig, Germany; 2 Department of Orthopaedic, Trauma and Plastic Surgery, University Hospital Leipzig, 04103 Leipzig, Germany

**Keywords:** Hajdu-Cheney Syndrome, NOTCH2 gene, autosomal dominant, hydrocephalus, basilar impression

## Abstract

**Background:** Hajdu-Cheney Syndrome is an autosomal dominantly inherited disease, with less than 50 patients reported to date. It is associated with gain-of-function variants of the NOTCH2 gene on chromosome 1p12. **Methods:** Here we present a case of NOTCH2 gene-associated Hajdu-Cheney syndrome with a progressive basilar impression and consecutive hydrocephalus. While the neuropsychologic development of the patient remained uneventful, allowing him to obtain his college exam, neurosurgical and orthopedic interventions became necessary to treat basal invagination and hydrocephalus at the age of 13 years. **Results:** Finally, the progressive compression of the medulla oblongata led to respiratory problems with the need for tracheotomy. The patient succumbed to his disease at the age of 18. **Conclusions:** To our knowledge, this is the first case in which a combination of hydrocephalus and basilar impression lead to a fatal outcome in spite of preemptive surgical interventions.

## 1. Introduction

The clinical features of Hajdu-Cheney syndrome are cranial and facial deformities, including musculoskeletal abnormalities, most commonly affecting the hands and fingers, and less commonly heart or kidney disease [1]. Half of patients with Hajdu-Cheney syndrome have mild facial anomalies in infancy, such as a small lower jaw. The remainder have an unremarkable course in infancy. With increasing age, additional phenotypic abnormalities such as facial dysmorphia occur. Delayed closure of the fontanelles is an associated symptom. Compression of the spine and osteolytic changes lead to progressive short stature. Acroosteolysis manifests in infancy with short fingertips and fingernails and pseudonodules on the fingers. Osteolytic activity leads to osteoporosis with biconcave vertebrae and kyphoscoliosis in childhood. Osteolysis at the transition from the skull to the cervical spine leads to basilar invagination, resulting in syringomyelia and hydrocephalus. Patients also have dental abnormalities with periodontitis and premature tooth loss. Some patients are at risk of renal failure due to multiple renal cysts. Serpentine fibula-polycystic kidney syndrome (SFPKS) is the most severe form of Hajdu-Cheney syndrome and is characterized by curved (serpentine) fibulae and radii and congenital renal cysts [2].

The disease is caused by a heterozygous mutation of the NOTCH2 gene, located on chromosome 1p12, which encodes the neurogenic Notch homologue protein 2 (Notch2). All pathological variants are located in exon 34 and result in increased NOTCH2 activity (gain of function) [3]. Increased function of the NOTCH2 gene may also lead to Alagille syndrome type 2, which is mainly a hepatic cholestatic disorder [4]. Diagnosis of Hajdu-Cheney Syndrom is primarily based on clinical features and symptoms, followed by radiological evaluation. Molecular pathology to prove mutation in the NOTCH2 gene is required for a definitive diagnosis, since the facial anomalies in infancy and early childhood can be misdiagnosed as Noonan Syndrome [5]. Prenatal testing is possible, and is advised to be carried out in families with Hajdu-Cheney syndrome, testing for chromosome 1p12 exon 34 mutations in the NOTCH2 gene.

Hajdu-Cheney syndrome is typically inherited as an autosomal dominant trait, meaning offspring have a 50% risk of developing the disease. However, in most cases, the syndrome occurs sporadically. Due to the rarity of Hajdu-Cheney syndrome, there are currently no treatment recommendations. Patients should be treated according to their symptoms. Bisphosphonates are used to treat osteoporosis, while standard therapy is recommended for patients with nephrological abnormalities. Regular dental and cardiologic examinations are important components of symptom-based management [1].

It should be noted, that in most cases life expectancy is not significantly affected by these conditions. Osteoporosis and associated fractures cause significant morbidity, while basilar invagination can occasionally lead to syringomyelia and rarely to hydrocephalus. Dental management is also important for quality of life. In severe cases, patients with Hajdu-Cheney syndrome suffer from bone abnormalities such as fibular deformities, hip joint atrophy, dental and jaw malocclusions, arachnodactyly, severe osteoporosis and osteolytic fractures [6]. Hajdu-Cheney syndrome can show a broad spectrum of clinical manifestations and, most importantly, displays an age-related disease progression of phenotypic features (Table 1).

## 2. Clinical Case

The patient was the only child of unrelated parents. Genetic confirmation of Hajdu-Cheney syndrome was performed at the age of 11. Clinically and neurologically, the patient had the following features due to Hajdu-Cheney syndrome: craniofacial dysmorphia, hydrocephalus occlusus, macrocephaly, Chiari malformation, bilateral cystic kidneys, short stature, bilateral hearing loss and hypogonadotropic hypogonadism.

The patient had been initially presented to the pediatric department at the age of 12 years, with the diagnosis of hydrocephalus. Placement of a ventriculo-peritoneal (VP) shunt system was then performed.

**Figure 1:**
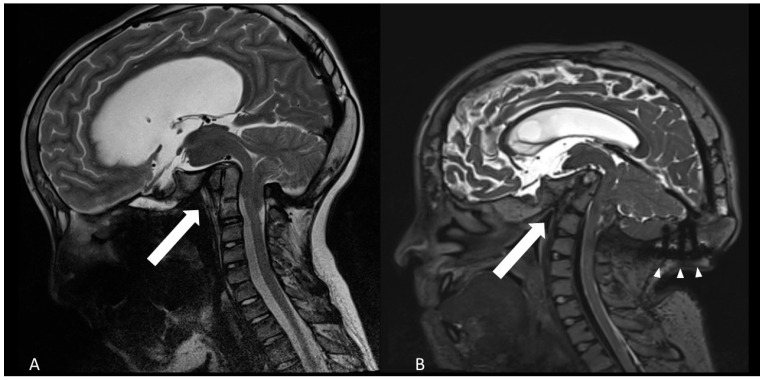
Progressive basilar invagination and flattening of the calvaria as shown by sagittal T2-weighted MRI. (**A**): age of 12 years (**B**): 18 years old. The arrows point to the angle between clivus and dens axis, diminishing from about 75° to 50°. Arrowheads show the occipital instrumentation.

**Figure 2:**
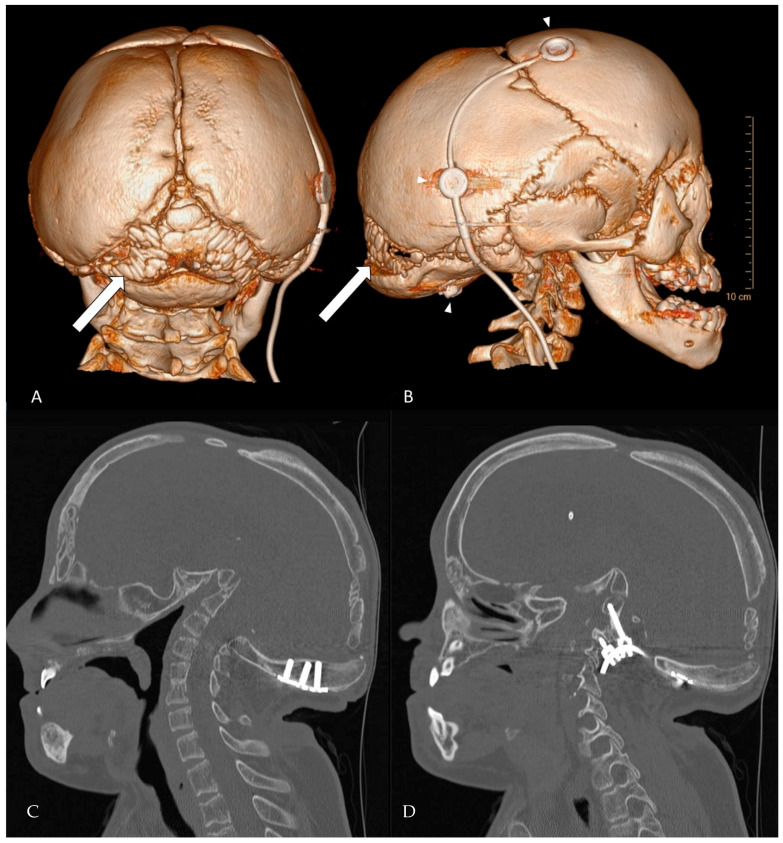
CT-rendering of Wormian bones at the age of 18 years. (**A**) posterior view of the skull. (**B**) lateral view. The arrows point to multiple intrasutural bone fragments arising in the lambdoid suture, a fragment can be seen in the coronal suture and pterion ossicles are present. The upper two arrowheads depict the VP-shunt system, the lower arrowhead points to a screwhead of the occipital instrumentation. (**C**,**D**) Complementary 2D bone-window CT slices (sagittal plane) detailing instrumentation the position and anatomy that are difficult to appreciate on 3D views. (**C**) Mid-sagittal slice showing the relationship of the occipital plate/screws to the foramen magnum and the cranio-cervical junction. (**D**) Left-parasagittal slice highlighting the occipito-cervical instrumentation and screw trajectory.

The patient’s clinical condition was significantly associated to basilar impression, as confirmed by the subsequent imaging studies (Figure 1). One year after VP-shunt insertion, the progressing basilar invagination had to be treated by decompression of the foramen magnum and fusion and decompression of C0–C2 (Figure 2). At age 13, the patient underwent posterior cranio-cervical surgery consisting of foramen magnum decompression in combination with C0–C2 decompression and occipito-cervical fixation spanning C0–C2. The decompression targeted the foramen magnum and the cranio-cervical junction to relieve brainstem compression. Fixation bridged the occiput to C2 to counteract progressive basilar invagination.

Still then, the radiologic controls disclosed a progressive basilar impression due to dysmorphic deformation of the head with dorsal tilt and a pronounced clivus deformation. Concordingly, a dual energy X-ray absorptiometry showed a Z-score of −4.4 in the area of the hips. Thus, the administration of bisphosphonates was initiated. Bisphosphonate therapy was started after identifying severe low bone mass (hip DXA Z-score −4.4). However, no standardized post-treatment DXA was obtained before the patient’s death and no serial long-bone radiographs were aquired to assess metaphyseal sclerotic “zebra lines”. Consequently, a treatment effect could not be quantified in this case.

When the first signs of brainstem compression occurred, a strong advice for a combined surgical approach had been given. The patient managed in the meantime to pass his college exam, and agreed to undergo the multidisciplinary operative intervention after finishing school. After a delay of more than 2 years and repeated follow-up consultations, counseling and radiological examinations, the parents and the patient finally chose a surgical procedure, already at a state of severe respiratory worsening. The patient was admitted as an in-patient with obstructive airway syndrome, neurological respiratory dysregulation, dysphagia and hoarseness.

Compared with previous images, the radiological studies showed a continuing ascending tip of the dens axis with bending of the brainstem and folding of the angle between clivus and dens (Figure 1).

In view of the clinical symptoms and as a consequence of the progressive respiratory dysfunction the further operation was offered as a rescue attempt. The aim was to stepwise stabilize and decompress the basilar impression on the brain stem. After preparing the occipital skull for screw support, unilateral removal of the former instrumentation followed by HALO extension was planned, then followed by a renewed occipitocervical spondylodesis.

Given progressive respiratory dysfunction and radiological progression with dens ascent and brainstem bending a staged rescue strategy was indicated. After preparing the occipital skull for future screw support, we planned unilateral removal of the previous occipital instrumentation, halo distraction and once stabilized we performed occipito-cervical spondylodesis. As the first stage, we performed preparatory cranial vault stabilization to enable secure halo traction and subsequent long-segment fixation. The preparative surgery was performed at the age of 18 years, and involved closure of the cranial grooves as an individualized craniodesis procedure. After a first step tracheotomy to secure the airways, the fontanel, calotte and sutures were exposed. The coronal and sagittal sutures were augmented with bone morphogenetic protein (BMP) paste and covered with a 3-dimensional titanium mesh on both sides up to the pterion. The lambdoid suture and occipital bone were left unfixated to enable mobilization during subsequent halo distraction, scheduled after wound healing and suture closure. This stage was completed as planned. Halo distraction and planned revision of occipito-cervical spondylodesis were not undertaken before discharge. The aims were fusion and stabilization of the cranial vault to prepare for HALO screw support, and application of distraction, before implanting a long-distance craniothoracal spondylodesis.

The patient was then transferred to the intensive care unit and recovered quite well after the operation. He was discharged home upon his parents’ request, despite the skull still being soft and tender without strong ossification. In the subsequent course, the patient and his family did not attend the follow-up appointments and declined further interventions. The patient succumbed to his disease about 8 weeks after the operation. The family did not wish an autopsy.

## 3. Discussion

Due to the rarity of Hajdu-Cheney syndrome, and the often-benign course, no clear treatment guidelines or recommendations exist. In function of the phenotypic expression, most treatment decisions respond to occurring symptoms. Many patients present with osteoclastic dysmorphia, sometimes associated to cardiac or neurological symptoms. The acroosteolysis with osteoporosis and subsequent changes of skull, mandible and thorax can lead to locomotor and respiratory issues. Given the rarity of HCS, we summarized representative published cases and contrasted them with our patient (Table 2).

To date, mutation in the NOTCH2 gene has been identified. Other molecular mechanisms and signaling pathways are currently unknown and require further intensive research. In most cases, overexpression of the NOTCH2 gene leads to increased osteoclastic activity and thus to a dysfunction of osteoblastic functions in bone.

Hajdu-Cheney syndrome is associated with Wormian bones, which are indicative of osteolytic activity in the skull (Figure 2). The severity of the disease has been correlated to the number of Wormian bones. Additionally, molecular genetic testing is essential for accurate diagnosis. Given the age-dependent evolution of HCS and the potential for cranio-cervical progression, we recommend structured monitoring: (i) baseline MRI of the cranio-cervical junction at diagnosis, (ii) repeat imaging every 12–24 months during growth, or earlier if new symptoms arise (e.g., dysphagia, stridor, sleep-disordered breathing, scoliosis acceleration) and (iii) dynamic cervical radiographs or CT when instability is suspected. Routine clinical follow-up should include neurological examination, respiratory assessment and periodic bone health evaluation. A low threshold for early referral to a cranio-vertebral junction team is warranted when radiographic progression or brainstem compression is documented. Early in life, facial features may overlap (short stature, facial dysmorphism), which can delay recognition of HCS. Practical clues favoring HCS include progressive acroosteolysis, Wormian bones and delayed suture closure, cranio-cervical osteolysis with basilar invagination, generalized osteopenia and osteoporosis. By contrast Noonan syndrome typically shows characteristics cardiac lesions and lacks progressive distal acroosteolysis. In uncertain cases, targeted NOTCH2 testing confirms HCS, whereas multigene RAS-MAPK panels support or exclude Noonan spectrum disorders. This distinction matters because surveillance priorities diverge (cranio-cervical stability and bone health in HCS versus cardiologic focus in Noonan [5,8,10].

It is not possible to predict the clinical outcome, due to a lack of molecular biomarkers or longitudinal studies. Life expectancy correlates with the clinical course and the severity of neurological symptoms, including basal brainstem impression with respiratory failure, as seen in our case [7]. HCS arises from heterozygous truncating variants in NOTCH2 gene, leading to increased NOTCH signaling and unbalanced bone remodeling with enhanced osteoclastic activity. At the cranial vault and skull base, persistent suture patency with Wormian bones reflects disturbed ossification, progressive osteolysis at the cranio-cervical junction with basilar invagination [5]. In our patient, this cascade with open sutures, vault deformity and cranial base resorption leads to medullary compression and central respiratory failure despite prior VP-shunting and occipito-cervical intervention. Understanding this sequence highlights the rationale for early stabilization strategies when progressive invagination is documented and adjuvant bone-active therapy to improve and surgical substrate.

Further symptoms, such as cardiac complications and vertebral fractures due to osteoporosis, can also affect quality of life and life expectancy. Due to the complex and varied constellation of symptoms, it is essential that detailed perioperative planning, post-operative management and close monitoring are carried out. To the best of our knowledge, we present the first case of a child with a combination of complex pathologic progression, obstructive hydrocephalus and basilar impression, leading to a fatal outcome. Of note, neurocognitive development had not been impaired, and signs of hepatic cholestasis, e.g., Alagille syndrome 2 were not detected.

This case report highlights the importance of interdisciplinary collaboration and prompt initiation of therapy upon diagnosis in Hajdu-Cheney syndrome. Specifically, the management of long-term osteolysis is a crucial aspect.

It has been suggested that early surgical closure of bone sutures can prevent the tearing of delayed-closing sutures. Long-segment spondylodesis can lower the rate of clinical deteriorations, unlike short-segment spondylodesis. Additionally, preoperative drug therapy for bone quality can significantly improve surgical treatment and postoperative outcome. In a case report, administration of romosuzumab over one year supported improvement of the bone mass index [11]. This might be a promising treatment option for osteoporosis in patients with Hajdu-Cheney syndrome.

Based on the literature, together with the present report, the lesson of this case is that the poor bone quality and the open sutures were the cause of an increasing speed of deterioration, in spite of the still expected adolescent growth [9]. Retrospectively, surgical closure of the sutures and early osteoporosis therapy, with an additional endocrinologic medication to support the applied bisphosphonates, might have slowed the osteoacrolysis.

## 4. Conclusions

This case illustrates how open sutures and skull-base osteolysis in HCS can accelerate cranio-cervical failure and, in extreme cases, lead to fatal respiratory compromise despite staged interventions. In our patient, bisphosphonate therapy was initiated for severe low bone mass, but treatment efficacy could not be documented (no follow-up DXA or metaphyseal “zebra lines” before death). We advocate structured childhood surveillance for basilar invagination (baseline MRI at diagnosis, re-imaging every 12–24 months during growth, and earlier if symptomatic) with a low threshold for early multidisciplinary evaluation when progression is detected. The timing of stabilization and the use of bone-active agents should be individualized and discussed within an experienced cranio-vertebral junction team.

## Figures and Tables

**Table 1:** Synopsis of reported symptoms in Hajdu-Cheney syndrome.

Organ System	Clinical Characteristics
Cranial	bathrocephaly, presence of multiple Wormian bones, delayed suture closure, thickened dome of the skull, absent frontal sinuses, elongated sella turcica, small jaw, basilar invagination, dolichocephaly, occipital prominence
Facial	coarse and dysmorphic facies, elongated philtrum, micrognathia, low-set ears, telecanthus, synophrys, bushy eyebrows, long eyelashes, wide nose, high arched palate, premature denture loss, jaw malocclusion, hirsutism, hypertelorism
Musculoskeletal	short stature, short neck, fractures of long bones, joint laxity, biconcave vertebrae, kyphoscoliosis, cervical instability, vertebral collapse, genu valgum, serpentine fibula, acroosteolysis, pseudoclubbing, short fingers, progressive distal bone resorption, bone demineralization, osteopenia, osteoporosis
Cardiovascular	congenital heart disease, patent arterial duct, septal defect
Intestinal	digestive alterations, intestinal malrotation, hernias
Neurologic	hydrocephalus, lateral meningocele
Urological	hypospadia, cryptorchidism, renal cysts, renal failure
Respiratory	thoracic deformities, ventilatory restriction, recurrent infections
Further alterations	delayed motor development, hearing loss, changes of the voice, deep voice, short nails, plantar ulcers

**Table 2:** Selected literature on Hajdu–Cheney syndrome (HCS) and related cranio-cervical management, contrasted with the present case.

Author and Year	Clinical Features	Interventions/Therapies	Outcome/Specific Relevance
Brennan & Pauli, 2001 [7]	Progressive skeletal resorption, variable cranio-cervical involvement reported across cases	Supportive and individualized management	Phenotypic evaluation over time; highlights surveillance needs in adolescence/adulthood
Cortés-Martín et al., 2020 (systematic review) [1]	Summarizes reported HCS manifestations; cranio-cervical disease variably present; hydrocephalus reported in some cases	Heterogeneous, case based	Aggregates the published experience to date, underlines rarity and lack of guidelines
Drake et al., 2003 [8]	Severe osteoporosis	Romosozumab for 12 months	Bone mineral density improvement, emerging adjunct for skeletal fragility in HCS
Cutler et al., 2023 [9]	Basilar invagination with open sutures	Cranial vault suspension	Technique relevant where suture patency complicates basilar invagination conceptually aligned with our preparatory craniodesis stage.
Present case	Basilar invagination and obstructive hydrocephalus, progressive brainstem compression	VP shunt, foramen magnum C0–C2 decompression and fixation, later preparatory cranial vault stabilization	Fatal central respiratory failure; illustrates rapid cranio-cervical progression despite staged surgical strategy.

## Data Availability

De-identified information may be available from the corresponding author upon reasonable request.
